# Analysis of Palatine and Adenoid Calcification in a Sample of the Lebanese Population Using Cone‑Beam Computed Tomography Technology

**DOI:** 10.7759/cureus.11238

**Published:** 2020-10-29

**Authors:** Raymond Challita, Sayde Sokhn, Ronald Challita, Anthony Challita, Georges Aoun

**Affiliations:** 1 Plastic and Reconstructive Surgery, Faculty of Medicine, Lebanese University, Beirut, LBN; 2 Oral Medicine and Maxillofacial Radiology, Faculty of Medicine, Lebanese University, Beirut, LBN; 3 Obstetrics and Gynecology, Faculty of Medicine, Lebanese University, Beirut, LBN; 4 Periodontology and Implantology, Faculty of Medicine, Lebanese University, Beirut, LBN

**Keywords:** tonsilloliths, palatine tonsils, adenoid tonsils, head and neck calcifications, cone-beam computed tomography (cbct)

## Abstract

Objectives

Tonsilloliths and adenoid calcifications are usually an incidental finding in radiologic studies. Several studies were done to evaluate the presence of tonsillar calcifications using different radiological techniques that include panoramic radiographs, computed tomography (CT), and cone-beam computed tomography (CBCT). These percentages varied in different populations and changed with the technique used. A CBCT is used to allow the visualization of the calcifications to make a better diagnosis. To the best of our knowledge, this is the first study conducted in Lebanon to study tonsillar and adenoid calcifications using CBCT. This study aims to assess the epidemiology and the demographic criteria of tonsilloliths and adenoid calcifications in a sample of the Lebanese population using the CBCT.

Methods

In this retrospective study, CBCT images of 125 patients attending a private clinic in Jbeil, Lebanon from May 2016 to July 2019 were selected. Images were evaluated by a maxillofacial radiologist. Adenoid and palatine calcifications were recorded. Statistical analysis was conducted using the Statistical Package for Social Sciences (SPSS) software, version 21 (IBM SPSS Statistics, Armonk, NY, USA). The level of statistical significance was established at a p-value < 0.05.

Results

A total of 21 patients (17.35%) had palatine calcifications and two patients (1.65%) had adenoid calcifications. From those with tonsilloliths, 12 female patients (16.43%) and nine male patients (18.75%) had palatine calcifications. Moreover, one male (2.08%) and one female (1.36%) had adenoid calcifications. There was no statistically significant difference in the distribution of calcifications with respect to gender. There was a non-statistically significant difference in the mean age of the patients with respect to the distribution of palatine and adenoid calcifications. The pattern of palatine calcifications showed a statistically significant difference in various age groups. Palatine calcifications distribution, with respect to the side, were statistically significant.

Conclusion

The prevalence of tonsilloliths in our sample was found to be 17.35%. The high prevalence detected was due to the technique used, the CBCT, which ensured a more detailed examination. The distribution was not affected by gender and age. However, multiple patterns of calcification were identified in younger age groups as opposed to other studies. Moreover, adenoid calcifications were present in 1.65% of the sample.

## Introduction

Tonsilloliths, also known as tonsil stones or tonsillar calcifications, are foul-smelling lumps. Their color can be yellow or white. They can be soft but can also be as hard as stones. They are formed of different calcium salts and can also contain ammonium radicals [1]. 

The palatine tonsils are one of the constituents of the Waldeyer’s ring. The other two components are the pharyngeal tonsils (adenoids) and the lingual tonsils [2]. 

Patients presenting with tonsilloliths may be symptomatic or not. It is usually an incidental finding on panoramic or lateral cephalometric radiographs. The superimposition in these radiographic techniques creates a diagnostic challenge. Several differential diagnoses are to be taken into consideration, such as sialoliths, tonsilloliths, phleboliths, calcified lymph nodes, and stylohyoid ligament ossification [3]. Therefore, cone-beam computed tomography (CBCT) is used to allow the visualization of the calcification in a three-dimensional (3D) image to make a better diagnosis [4].

Several studies were done to evaluate the presence of tonsillar calcifications using different radiological techniques that include panoramic radiographs, computed tomography (CT), and CBCT [1-2, 4-7]. The prevalence varied in different populations and changed with the technique used. It ranged between 5.7% and 46.1% with higher detection rates when using the CT or the CBCT.

Although tonsilloliths are relatively common, the presence of adenoid calcifications is rare [8]. Most of these lymphoid calcifications are asymptomatic, but they can cause pain and halitosis [9].

Hence, we conducted this study to assess the prevalence and the demographic criteria of tonsilloliths and adenoid calcifications in a sample of the Lebanese population using CBCT. To the best of our knowledge, this is the first study conducted in Lebanon to study tonsillar and adenoid calcifications using CBCT.

## Materials and methods

A cross-sectional retrospective study was conducted on a sample of the Lebanese population. A total of 125 CBCT images of patients attending a private clinic in Jbeil-Lebanon from May 2016 to July 2019 were analyzed.

Inclusion criteria included Lebanese patients with a CBCT done for multiple indications (e.g., impacted teeth, sinus diagnosis, implant planning). All of the images with motion artifacts were excluded. 

Patients were informed that the radiographs might be anonymously used for research purposes at a later stage and their consent was obtained (or a guardian for patients under 18). Moreover, due to the retrospective nature of this study, it was granted an exemption in writing by the Ethical Committee, Private Clinic Imaging Center, Jbeil, Lebanon.

Technique

The scans were acquired using two kinds of CBCT machines: the PaX‑Zenith3D^©^ machine (Vatech Co. Ltd., Yongin‑Si, Republic of Korea) and NewTom VGi (QR Srl Company, Verona, Italy). The technical parameters ranged between 70 and 100 kVp and 7 - 15 mA, with an exposure time of 20 - 35 s and a medium to a large field of view, according to the clinical case, with respect to the “as low as reasonably achievable.” The voxel was 0.4 mm. All images were then reconstructed in axial and coronal sections, having both a 0.4 mm thickness and interval.

Next, the CBCT images were evaluated by an experienced maxillofacial radiologist who was familiar with CBCT. Forty CBCT images were reviewed again by the same maxillofacial radiologist. The intraobserver agreement was 100% for the presence and patterns of calcifications. This individual evaluated the images to determine the presence of palatine and adenoid tonsil calcifications, their locations (unilateral or bilateral), and the form of calcifications (single or multiple).

Statistical analysis was conducted using the Statistical Package for Social Sciences (SPSS) software, version 21 (IBM SPSS Statistics, Armonk, NY, USA). The level of statistical significance was established at a p-value < 0.05.

## Results

**Figure 1:**
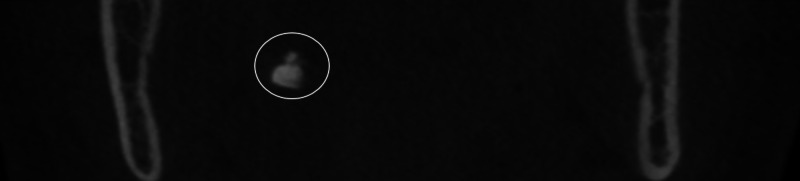
Tonsilloliths in a 60-year-old asymptomatic male A 60-year-old asymptomatic male who came for implant restorative treatment. The frontal cut of the CBCT of the mandible (FOV: 6 cm) shows single and unilateral tonsilloliths calcification (white circle). CBCT: cone-beam computed tomography; FOV: field of view

**Figure 2:**
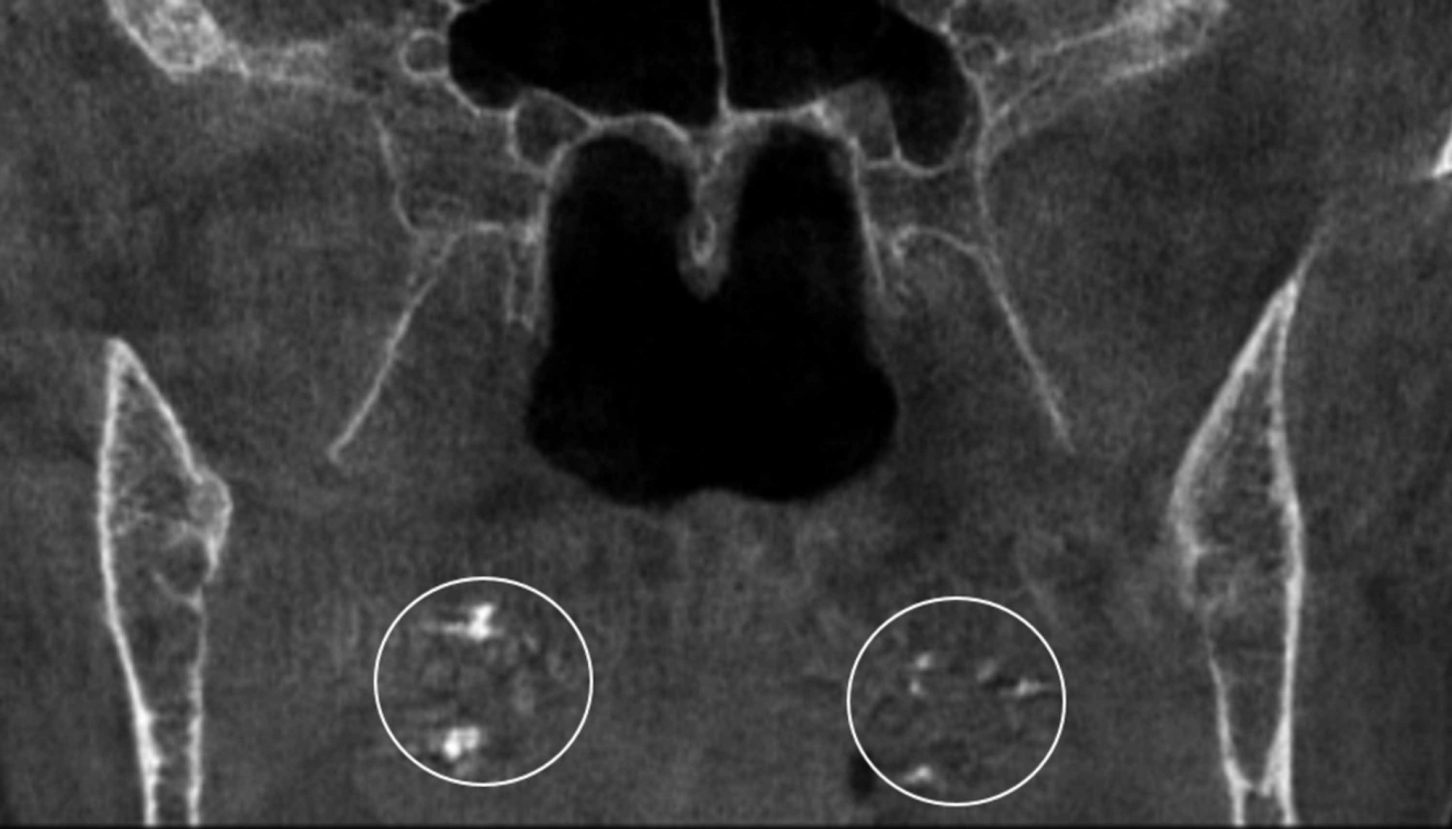
Multiple tonsilloliths in an 82-year-old female An 82-year-old female patient presenting for dental extraction. She had a history of recurrent tonsillar inflammation. A frontal cut of a CBCT, FOV: 8 cm (maxilla and mandible) shows multiple and bilateral tonsillolith calcifications (white circle). CBCT: cone-beam computed tomography; FOV: field of view

**Figure 3:**
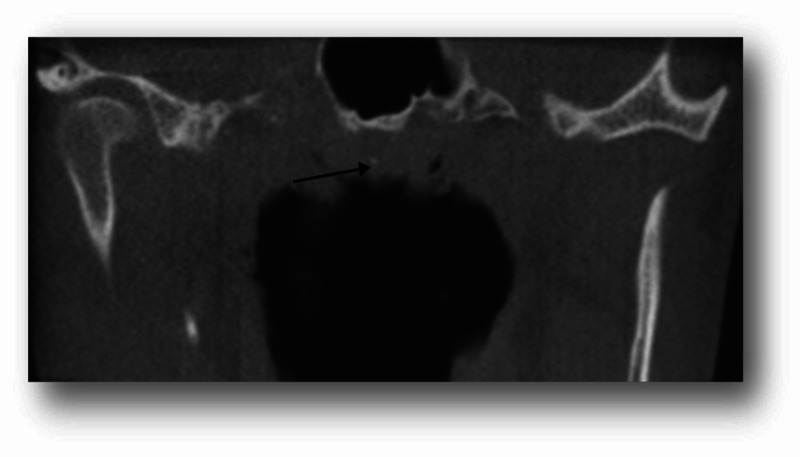
Adenoid calcification in a 43-year-old male A 43-year-old male patient presented for dental treatment (asymptomatic). A frontal cut of the CBCT, FOV: 6 cm (maxilla) showed a single adenoid calcification (black arrow). CBCT: cone-beam computed tomography; FOV: field of view

**Figure 4:**
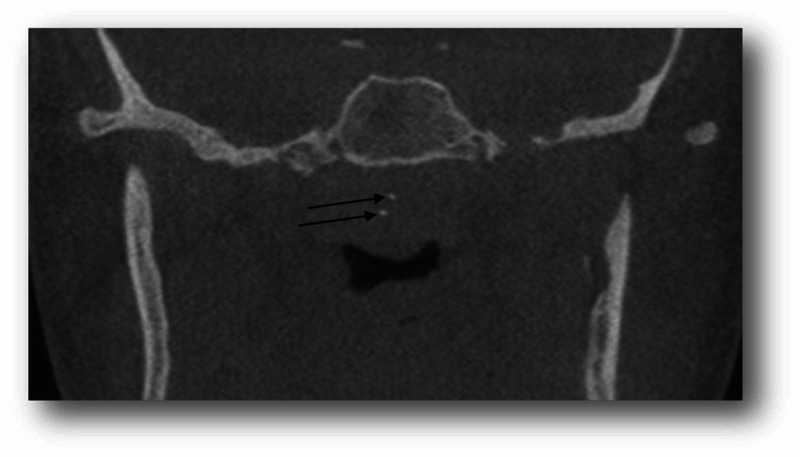
Adenoid calcifications in an 18-year-old female An 18-year-old female patient (asymptomatic) presented for dental treatment. A frontal cut of the CBCT, FOV: 8 cm (maxilla and mandible) showed multiple adenoid calcifications (black arrows) CBCT: cone-beam computed tomography; FOV: field of view

After the exclusion of images with motion artifacts, a total of 121 CBCT images (73 females and 48 males) were reviewed in this study. The age of the patients ranged between 14 and 82 years with a mean of 40.95 ± 16.79. Twenty-one patients (17.35%) had palatine calcifications and two patients (1.65%) had adenoid calcifications. Of these patients, 16 patients (13.22%) had single palatine calcifications (Figure 1), five patients (4.13%) had multiple palatine calcifications (Figure 2), one patient (0.82%) had single adenoid calcification (Figure 3), and one patient (0.82%) had multiple adenoid calcifications (Figure 4). Of note, in our sample, no patient had associated adenoid and palatine calcifications.

The mean age of patients with palatine and adenoid calcifications are represented in Table 1. Concerning gender, 12 female patients (16.43%) and nine male patients (18.75%) had palatine calcifications. Moreover, one male (2.08%) and one female (1.36%) had adenoid calcifications. There was no statistically significant difference in the distribution of calcifications with respect to gender for palatine (p = 0.743) and adenoid calcifications (p = 0.336). Moreover, there was no statistically significant difference in the pattern of calcifications (single or multiple) for adenoid and palatine calcifications (p = 0.375) with respect to gender (Table 1).

**Table 1:** The Distribution of Palatine and Adenoid Calcifications With Respect to Gender Abs: absent; F: female; M: male

		Abs palatine	Single palatine	Multiple palatine	Abs adenoid	Single adenoid	Multiple adenoid
Gender	F	61	9	3	72	0	1
	M	39	7	2	47	1	0

With respect to age, the mean age of patients with calcifications is represented in Table 2. There was no statistically significant difference in the mean age of the patients with respect to the distribution of palatine and adenoid calcifications. The distribution of palatine (p = 0.312) and adenoid calcifications (p = 0.675) was not statistically significant in various age groups (Table 3). However, the pattern of palatine calcifications showed a statistically significant difference in various age groups (p = 0.001) (Table 4). In our sample, 11 patients (9.1%) had right side palatine calcifications, six patients (5%) had left-sided palatine calcification, and five patients (4.1%) had both right and left side calcifications. These values were statistically significant with respect to the side (p-value: 0.001). Of note, on both sides, the most common pattern of calcification was the single pattern.

**Table 2:** Mean Age of Patients With and Without Palatine and Adenoid Calcifications Abs: absent

	Mean	#	Standard Deviation
Abs Palatine Tonsils	39.86	100	16.685
Multiple Palatine Tonsils	41.80	5	28.499
Single Palatine Tonsils	47.50	16	12.171
Abs Adenoid Calcification	41.14	119	16.776
Single Adenoid Calcification	43.0	1	
Multiple Adenoid Calcification	16.00	1	

**Table 3:** The Distribution of Palatine and Adenoid Calcifications in Various Age Groups

		Age group							
		Under 20	20-29	30-39	40-49	50-59	60-69	70-79	≥ 80
Palatine	Absence	15	21	11	18	21	13	1	0
	Presence	1	4	1	4	7	3	0	1
Adenoid	Absence	15	25	12	21	28	16	1	1
	Presence	1	0	0	1	0	0	0	0

**Table 4:** Pattern of Palatine Calcifications in Various Age Groups

		Age Group								
		under 20	20-29	30-39	40-49	50-59	60-69	70-79	≥ 80	Total
Palatine tonsils (single or multiple)	Abs	15	21	11	18	21	13	1	0	100
	Single	1	1	1	4	7	2	0	0	16
	Multiple	0	3	0	0	0	1	0	1	5
Total		16	25	12	22	28	16	1	1	121

## Discussion

Maxillofacial calcifications are mostly incidental findings on digital panoramic radiographs (DPR) or CBCT. They can occur in various structures and are mainly associated with chronic inflammation. Many studies in the literature have tried to describe the characteristics of these calcifications. These mainly include tonsilloliths, calcified lymph nodes, atheromas, calcified styloid processes, phleboliths, and calcified laryngeal cartilages [10].

Tonsilloliths are uncommon calcifications in the maxillofacial area. They are incidental findings discovered on radiologic studies [11]. Their prevalence is variable with various studies indicating different percentages [12]. This may be due to the different imaging techniques used. They can be asymptomatic or can present with halitosis, cough, ear pain, and foul taste [11]. In Lebanon, no study was done to assess the prevalence of tonsilloliths using CBCT. The prevalence was only calculated using DPR, which may be responsible for the low detection rates in most circumstances [7]. Moreover, finding calcifications in the adenoid tissue is really rare. These calcifications are not frequently discussed in the literature. They are also usually associated with halitosis [8].

In the current study, the prevalence of tonsilloliths was 17.35%. Multiple studies in the literature noted various percentages. This prevalence ranged between 16% and 46.1% [13-15]. Lower percentages, such as 5.7% and 13%, were identified [3, 6-7, 11]. These differences are mainly due to the imaging technique used. DPR had the lowest detection rates, whereas CBCT and CT scans had better detection rates. These rates also change with the change in slice thickness [16]. A study by Aoun et al. conducted on the Lebanese population using DPR reported that tonsilloliths were present in 7.2% of the population [7]. However, in our sample, a prevalence rate of 17.35% can be expected. This is considered due to the technique used where the slice thickness used in the CBCT was 0.4 mm. This thickness increases the sensitivity and improves the detection rate. Therefore, better prevalence rates can be detected using CBCT. Of note, the demographics of the patients also affects the prevalence rate [12]. 

About 16.43% of the females and 18.75% of the male patients had tonsilloliths. However, a non-statistically significant difference was present with respect to gender. This is similar to a study performed on the Lebanese population using DPR [7]. Most of the studies in the literature showed no relationship between gender and the prevalence of tonsilloliths [1, 14-15]. However, in a study conducted in southern Iran, men were found to be 1.7 times more likely to develop tonsil calcifications when compared to women [17]. Gender may not be considered a risk factor for the development of tonsilloliths in the Lebanese population. The pattern of tonsillar calcification was also not affected by gender. This finding is similar to the study performed by Kajan et al. [4].

Concerning age, the mean age of patients with single calcifications in our study was 47.5 ± 12.17, with multiple calcifications 41.8 ± 28.49, and with no calcifications 39.86 ± 16.68. There was a non-statistically significant difference between these groups. Moreover, 33.33% of the tonsilloliths were found in the age group of 50 - 59 years but a statistical significance was not found (p = 0.312). This is in contrast to an Austrian study where most of the calcifications were present in patients older than 40 years [6]. Various studies showed that tonsilloliths were mainly detected in patients over 40 years of age with increasing incidence in older ages due to the increased risks of recurrent inflammatory processes [9, 14]. However, no age predilection was present in the current study consistent with the study already performed on the Lebanese population by Aoun et al. where a poor relation was observed between tonsilloliths and age. This was also evident in a study by Fauroux et al. where no relation was found between age and tonsilloliths [15]. The majority of single calcifications were found in the 50 - 59 age group, whereas multiple calcifications were found in the 20 - 29 age group. These findings were statistically significant. Chronic and recurrent infections can lead to tonsilloliths. The presence of multiple calcifications and the prevalence of tonsilloliths is more common in the elderly [18-19]. This was not the case in our study, where most of the multiple calcifications were found in patients younger than 30 years. This may be due to multiple recurrent inflammations occurring in the young Lebanese population. A further study is needed to assess why this age group has more multiple calcifications.

Most of the patients in our study had single palatine calcifications; however, 4.13% of the patients had multiple calcifications. Tonsilloliths are usually single [20]. According to Bamgbose et al., 62.90% of the tonsilloliths were multiple calcifications [3]. 

According to Ram et al., tonsilloliths occur more frequently on the right side [19]. This was also present in a study conducted by Bamgbose et al. [3]. However, Oda et al. reported no significant difference in the prevalence of tonsilloliths with respect to the palatine side [14]. However, in our study, tonsilloliths were more frequently detected on the right side and the single type calcification was the prevalent type on both sides.

The prevalence of adenoid calcification was reported to be 6% [9] and 12.9% [4]. However, in our sample, only 1.65% of the patients had adenoid calcifications. This value is small when compared to other studies. Moreover, none of the patients had both palatine and adenoid calcifications in contrast to other studies where various percentages of both calcifications were found [4]. Adenoid calcifications distribution was not affected by age or gender in our study. Females and males were equally affected. Chronic inflammation may also be the reason behind adenoid calcifications [8]. Consequently, increasing age would be a risk factor for developing these calcifications. However, this was not the case in our sample. Similarly, Kajan et al. showed that adenoid calcification was not affected by gender [4].

Concerning the limitations of the study, our sample was a small sample with the need for a larger one in the future to study the characteristics of palatine and adenoid calcifications. Moreover, our sample did not contain the same number of males and females.

## Conclusions

The prevalence of tonsilloliths was found to be 17.35% in our sample. CBCT ensures a more detailed examination which improves detection rates and increases the prevalence. Tonsillolith distribution was not affected by gender and age. However, multiple patterns of calcification were identified in younger age groups. Adenoid calcifications were present in 1.65% of the sample.
